# Drawing Teeth in Africa

**Published:** 1844-12

**Authors:** 


*Drawing Teeth in Africa.*—Every man in Africa is his own dentist, says the Liberia Herald. When a person wishes to get rid of a bad tooth, it is brought about in the following manner. A fine, strong cord is made of the fibres of the palm leaf—one end of which is fastened securely round the defective tooth. To the other end is tied a stone, weighing from eight to ten pounds—which is lifted up at arms' length and then let fall. The troublesome member is out in a twinkling—much quicker, at all events, than it could be extracted by any instrument, in civilized hands—and that is the end of the operation.

